# Uric acid is associated with microalbuminuria and decreased glomerular filtration rate in the general population during 7 and 13 years of follow-up: The Tromsø Study

**DOI:** 10.1186/s12882-015-0207-1

**Published:** 2015-12-11

**Authors:** Hilde M. Storhaug, Ingrid Toft, Jon Viljar Norvik, Trond Jenssen, Bjørn O. Eriksen, Toralf Melsom, Maja-Lisa Løchen, Marit Dahl Solbu

**Affiliations:** Department of Hematology, University Hospital of North Norway, N-9038 Tromsø, Norway; Metabolic and Renal Research Group, UiT The Arctic University of Norway, Tromsø, Norway; Department of Nephrology, University Hospital of North Norway, Tromsø, Norway; Department of Nephrology, Rikshospitalet, University Hospital, Oslo, Norway; Department of Community Medicine, UiT The Arctic University of Norway, Tromsø, Norway

**Keywords:** Serum uric acid, Renal dysfunction, Epidemiology, Albumin-creatinine ratio

## Abstract

**Background:**

The role of uric acid in development of renal dysfunction (RD) remains controversial. Earlier studies have reported inconsistent results, possibly because of their varying ability to adjust for confounding. The impact of longitudinal change in uric acid on renal outcome has not been assessed previously. We aimed to study the impact of change in serum uric acid (SUA) as well as baseline SUA on the development of RD.

**Methods:**

In a prospective cohort study, we assessed the associations between change in SUA during follow-up, baseline SUA and RD (defined as albumin-creatinine-ratio (ACR) ≥1.13 mg albumin/mmol creatinine and/or eGFR < 60 ml/min/1.73 m^2^) in a large cohort from a general population participating in the Tromsø Study (*n* = 2637). Participants were stratified according to tertiles of change in SUA between baseline (1994/95) and follow-up 13 years later. (upper tertile: SUA increasing group, two lower tertiles: SUA non-increasing group). Logistic regression analysis was applied with RD and each component of RD after 7 and 13 years as the dependent variables. Adjustments were made for baseline eGFR, cardiovascular risk factors, and the use of antihypertensive drugs including diuretics.

**Results:**

After excluding participants with RD at baseline, SUA increasers, compared to SUA non-increasers, had a doubled risk of RD after 7 years (odds ratio 2.00, (95 % CI 1.45, 2.75)). Odds ratio for RD in SUA increasers after 13 years was 2.18 (95 % CI 1.71, 2.79). The risk of developing ACR ≥1.13 mg/mmol alone was not significantly increased after 7 years (odds ratio 1.30 (95 % CI 0.90, 1.89), but after 13 years (odds ratio 1.43 (95 % CI 1.09, 1.86)). An increase in baseline SUA of 59 μmol/L (1 mg/dL) gave an odds ratio for RD after 13 years of 1.16 (95 % CI 1.04, 1.29).

**Conclusion:**

An increase in SUA during follow-up was associated with an increased risk of developing RD after 7 and 13 years.

## Background

Over the last few decades, chronic kidney disease (CKD) has emerged as a global health problem of epidemic proportions [1]. The prevalence of end-stage renal disease, and concomitantly the number of patients on renal replacement therapy, is steadily increasing, and these patients have a 10-fold mortality rate [2]. However, also lower stages of CKD have a significant impact on the population health. Estimated glomerular filtration rate (eGFR) less than 60 mL/min/1.73 m^2^ and urinary albumin creatinine ratio (ACR) of 1.13 mg/mmol or more are independent predictors of mortality risk in the general population [3]. We therefore contend that there is a need to search for more modifiable risk factors for CKD.

Hyperuricemia has been recognized as a risk factor for the incidence and progression of CKD, although studies have reported conflicting results [4]. Studying the role of SUA in CKD is difficult since uric acid is excreted primarily by the kidneys, and a decrease in GFR will be accompanied by a rise in SUA level. However, a Japanese study recently assessed the impact of SUA on the natural history of eGFR. The authors claimed that if elevation of SUA is a result, rather than a cause, of a decline in eGFR, the relationship between SUA and eGFR should be the same in the same population over years except for shifts by age-dependent reduction of eGFR. Multiple regression analyses showed that explanatory factors of eGFR after a 6-year interval were age and SUA at baseline. They concluded that elevation of SUA accelerated the yearly decline in eGFR [5].

Some evidence suggests that uric acid itself may be harmful in patients with CKD by contributing to increased inflammation and thus a progression in CKD [4]. Although controversial, these observations are supported by large prospective observational studies showing that increased SUA levels predict the development and progression of CKD in various populations [2, 6–11]. However, several of them do not have information on albuminuria as well as the use of allopurinol, diuretics and other medication which could confound the results. Furthermore, knowledge about the effect of change in SUA on renal function is scarce. The object of the present study was to assess whether increased SUA may predispose to the development of renal dysfunction (RD) in a prospective study in the general population. We also aimed to assess the possible impact of change in SUA during observation and examine the association between SUA and albuminuria.

## Methods

The Tromsø Study is an ongoing population based, prospective study with six repeated health surveys in the municipality of Tromsø, Northern Norway [12]. To the fourth survey (Tromsø 4) in 1994/95, all inhabitants aged 25 and above were invited, and 27,158 (77 %) participated. All participants aged 55–74 years, and 5–10 % of the other birth cohorts older than 24 years (10,542 individuals), were invited to a second visit with extensive examination including blood and urine testing. Attendance rate was 76 % (7965 individuals). Subjects who had previously taken part in the second visit of Tromsø 4 were eligible for a second-visit examination also in Tromsø 5 (2001/02), and 5939 subjects participated (85 %). Tromsø 6 was run in 2007/08. A total of 12,983 men and women aged 30–87 years participated, 65 % of all invited persons. Similar to Tromsø 4 and 5, a large sample from the Tromsø 6 cohort was invited to a second visit, and 7307 individuals attended. About 80 % of the participants in Tromsø 6 had attended Tromsø 4. SUA measurements were obtained from all of the three surveys. In Tromsø 4, SUA was measured in 6458 participants. Among these, SUA was measured in 4967 and 3047 persons in Tromsø 5 and 6, respectively. In this article, we describe the 2637 participants who had SUA measurements in all three surveys. All individuals with baseline measurements who died before Tromsø 6 or were lost to follow-up for other reasons were excluded. The Regional Committee for Medical Research Ethics (REC North) approved the study, and all participants gave informed written consent.

### Clinical and laboratory measurements

In each survey the participants responded to a self-administered questionnaire with information about current medication, history of cardiovascular disease and diabetes, smoking habits and physical activity. Height and weight were measured and body mass index (BMI) calculated [12]. Blood pressure was recorded with an automatic device (Dinamap Vital Sign Monitor 1846 Critikon). Three measurements were made at one minute intervals after 2 min of rest, and the mean of the two final recordings was used. Hypertension was defined as systolic blood pressure ≥ 140 mmHg and/or diastolic pressure ≥ 90 mmHg and/or current use of antihypertensive medication. Leisure physical activity was classified as active (>1 h physical activity with prominent sweating or breathlessness per week) or inactive (all others). Smoking habits were classified as non-smokers or current smokers. SUA was measured by photometry with COBAS® instruments (Roche diagnostics, Switzerland) using an enzymatic colorimetric test, the uricase/ PAP method. Reference values were140-340 μmol/L (2.45.7 mg/dl) for females and 200–415 μmol/L (3.4–7.0 mg/dl) for males. Change in SUA from 1994/95 to 2001/02 was calculated as SUA in Tromsø 5 minus SUA in Tromsø 4. Similarly, change in SUA from 1994/95 to 2007/08 was calculated.

In the U.S National Health and Nutrition Examination Survey (NHANES) 2007–2008, hyperuricemia was defined as SUA >417 μmol/L (7 mg/dL) in men and >339 μmol/L (5.7 mg/dL) in women, and the same definition was applied in the present study [11]. In Tromsø 4 and 5, serum creatinine was analyzed by a modified Jaffe reaction, but since creatinine-based estimation of GFR is better validated for enzymatic creatinine measurements, 111 plasma samples from Tromsø 4 and 142 samples from Tromsø 5 were thawed and reanalyzed with an enzymatic method (Modular P/Roche). Values were fitted to a linear regression model, and recalibrated creatinine values were calculated for all participants. In the Tromsø 6, serum creatinine was analysed on a Hitachi Modular model using an enzymatic method that has been standardized against isotope dilution mass spectroscopy (CREA Plus, Roche Diagnostics, GmbH, Mannheim, Germany). eGFR was calculated according to the CKD-EPI equation [13]. Three separate samples of morning spot urine from three consecutive days were collected and analyzed within 20 h. Urinary albumin and creatinine were analyzed using kits from ABX Diagnostics, Montpellier, France. ACR in mg albumin per mmol creatinine (mg/mmol) was calculated for each day and the mean of all three was used in the analyses. Serum cholesterol was analyzed by enzymatic colorimetric methods with commercial kits (CHOD-PAP; Boehringer Mannheim, Mannheim, Germany).

### Statistical analysis

Data are given as mean (±SD) for (normally distributed) variables or as median (interquartile range) for variables with a skewed distributions. Categorical variables are given as numbers (percentages). Baseline cardiovascular risk profile including current use of antihypertensive medication, diuretics, proportion of smokers, physical activity, BMI, blood pressure, SUA, creatinine, eGFR, and ACR was compared between the persons who only participated in the Tromsø 4 study and the participants who also attended follow-up in 2007/08 (Tromsø 6). Two-tailed independent groups t-tests were used for normally distributed variables and Mann-Whitney’s test otherwise. For categorical variables Chisquare tests were used. Change in SUA from 1994/95 to 2007/08 was categorized into tertiles. Tertile 1 and 2 were defined as SUA non-increasers, and tertile 3 was defined as SUA increasers. Several classical cardiovascular risk factors were compared between the two groups using independent groups t-tests, Mann-Whitney’s tests and chi-square tests as appropriate. The dichotomous variable RD was defined using a modification of the 2012 KDIGO CKD classification [14]. We chose the “high normal” albuminuria stage (ACR ≥ 1.13 mg/mmol) as the cut-off value for pathological urinary albumin excretion. Participants with eGFR < 60 ml/min/1.73 m^2^ and/or ACR ≥ 1.13 mg/mmol were considered to have RD. Logistic regression analyses were performed with this variable as the dependent variable. As independent variables, we included the upper versus the two lower tertiles of change in SUA from 1994/95 to 2007/08 (SUA increasers vs. SUA non-increasers), baseline values of SUA, eGFR, ACR, age, mean systolic pressure, smoking, diabetes, BMI, cholesterol, physical activity, and antihypertensive medication including diuretics. ACR was logarithmically transformed in order to achieve the best fit of the models. In the models, we also included change in physical activity, start of antihypertensive drugs, and change of smoking habits during observation time. We also performed logistic regression analyses with isolated ACR ≥ 1.13 mg/mmol or isolated GFR < 60 ml/min/1.73 m^2^ at follow-up as the dependent variables and the same independent variables as listed above. All analyses were done for the cohort followed from 1994/95 until 2001/02 and then for the same cohort followed from 1994/95 until 2007/08. First, the analyses were done in a subgroup that only included the participants who did not have RD at baseline. Finally, all the logistic regression analyses were repeated with the entire cohort included. The statistical analyses were run using SPSS software, version 21.0 (SPSS, INC., Chicago, Illinois) and a two-tailed *p* value <0.05 was considered statistically significant.

## Results

There were 2637 participants who had measurements of SUA from all three surveys, Tromsø 4, 5 and 6. Compared to the participants who attended one or more of the follow-up surveys, the participants who only attended the Tromsø 4 Study (1994/95) had a less favorable cardiovascular risk profile (data not shown). They were older, had higher SUA level, lower eGFR, higher ACR, higher BMI, and higher cholesterol. There were also more participants with hypertension, known diabetes, and a history of myocardial infarction and stroke in this cohort.

### Cohort characteristics

In the final cohort, 250 men (20 %) and 196 women (14 %) had hyperuricemia at baseline (Tromsø 4; 1994/95). Seven years later, in Tromsø 5, the corresponding percentages were were 16 % and 18 %, respectively, whereas 17 % of the male and 22 % of the female participants had hyperuricemia in Tromsø 6 (2007/08). At baseline, RD (defined as ACR ≥ 1.13 mg/mmol and/or eGFR < 60 ml/min/1.73 m^2^) was present in 422 (16 %) of the participants; 404 (15 %) had ACR ≥ 1.13 mg/mmol and 32 (1.2 %) had eGFR < 60 ml/min/1.73 m^2^. After 7 years (Tromsø 5) the number of participants with RD had increased to 474 (18 %). Among these, 394 (15 % of the total cohort) had ACR ≥ 1.13 mg/mmol and 117 (4 %) had eGFR < 60 ml/min/1.73 m^2^. The highest prevalence of RD was found in 2007/08 (Tromsø 6), when 697 (26 %) of the participants had the combined endpoint RD; ACR ≥ 1.13 mg/mmol was found in 589 (22 %) and decreased eGFR was present in 220 (8 %) of the participants.

In the entire cohort, SUA increased only slightly between the Tromsø 4 and Tromsø 6 studies; mean (± SD) of SUA change was 7 (±73) μmol/L. However, the large SD compared to the mean indicated a vast dispersion. Thus, the SUA change was dichotomized into SUA non-increasers (two lower tertiles of SUA change), and SUA increasers (upper tertile of SUA change). The cut-off between the SUA non-increasers and increasers was a SUA change during follow-up of 33 μmol/L. In the SUA non-increaser group, the mean SUA change over 13 years was −30 (±54) μmol/L, whereas the mean rise in SUA was 81 (±46) μmol/L in the SUA increaser group. During the first 7 years, the SUA increasers had a mean increase in SUA of 65 (±35) μmol/L whereas the corresponding 7-year change in SUA for the non-increasers was −24 (±49) μmol/L.

Table 1 shows characteristics at baseline (1994/95) and follow-up (2007/08) for the male SUA increasers vs. the SUA non-increasers. Table 2 shows the same variables at baseline and follow-up for the females. Both the baseline and follow-up clinical characteristics showed the same pattern in men and women. There was no significant difference in eGFR between SUA increasers and non-increasers at baseline. At follow-up eGFR had decreased in both groups, markedly more for SUA increasers. The proportion of participants with hypertension was higher among SUA increasers both at baseline and at follow-up. The use of diuretics was approximately the same in SUA increasers and non-increasers at baseline. At follow-up the use of diuretics was markedly higher among SUA increasers. Very few participants (0.2–0.8 %) were using allopurinol both at baseline and follow-up, and the use of allopurinol was not an independent predictor for any renal endpoint. The proportion of current smokers was similar between the two groups (women) or higher among SUA increasers than nonincreasers (men) at baseline, whereas the opposite was found at follow-up. There were slightly more females than males among SUA increasers. However, there was no significant interaction between gender and SUA change during follow-up for the prediction of RD. Therefore, the multivariable analyses were not run gender specific.

**Table 1:** Clinical characteristics at baseline and after 13 years of follow up of the male participants with and without increase in serum uric acid (SUA). The 4^th^ (1994/95) and the 6^th^ (2007/08) Tromsø Study (*n* = 1270)

	Baseline 1994/95 SUA non-increasers (*n* = 920)	SUA increasers (*n* = 350)	*p*-value*	Follow-up 2007/08 SUA non- increasers (*n* = 920)	SUA increasers (*n* = 350)	*p*-value**
Age (years)	56 ± 9	58 ± 8	<0.001	69 ± 9	71 ± 8	<0.001
SUA (μmol/L)	373 ± 81	320 ± 62	<0.001	330 ± 61	403 ± 77	<0.001
Estimated GFR (ml/min/1.73 m^2^)	96 ± 12	95 ± 11	0.17	85 ± 14	78 ± 17	<0.001
Annual change in estimated GFR (ml/min/1.73 m^2^)	-	-	-	−0.87 ± 0.71	−1.30 ± 0.93	<0.001
ACR (mg/mmol)	0.45 (0.33, 0.80)	0.52 (0.35, 0.86)	0.02	0.46 (0.22, 0.99)	0.60 (0.27, 1.49)	<0.001
Systolic blood pressure (mmHg)	139 ± 17	144 ± 19	<0.001	145 ± 21	148 ± 21	0.05
Hypertension, n (%)	464 (50)	216 (62)	<0.001	673 (73)	288 (82)	<0.001
Body mass index (kg/m^2^)	26.3 ± 3.0	25.9 ± 2.6	0.03	27.2 ± 3.5	27.4 ± 3.3	0.10
Cholesterol (mmol/L)	6.56 ± 1.19	6.50 ± 1.10	0.3	5.45 ± 1.16	5.40 ± 1.1	0.7
Current smokers, n (%)	239 (27)	112 (32)	0.03	144 (16)	36 (11)	0.04
Physical activity, n(%)	301 (33)	122 (35)	0.9	268 (29)	91 (32)	0.23
Antihypertensives incl. diuretics, n (%)	94 (10)	40 (11)	0.5	94 (10)	40 (11)	<0.001
Diuretics, n (%)	4 (0.3)	1 (0.3)	0.9	55 (6)	43 (13)	0.001
Diabetes, n (%)	14 (1.5)	5 (1.4)	0.9	116 (13)	43 (12)	0.9
Previous myocardial infarction and/or stroke, n(%)	66 (7)	21 (6)	0.5	-	-	-
Started antihypertensive treatment during the observation time, n (%)				279 (30)	158 (45)	<0.001
Stopped smoking during the observation time, n (%)				113 (12)	77 (22)	<0.001
Became physically active during the observation time, n (%)				126 (14)	41 (12)	0.4

Values are given as mean (standard deviation), median (interquartile range) and number (percentages) as appropriate. ACR: urinary albumin/creatinine ratio. *P*-values for differences between the SUA increasers and non-increasers at baseline* and follow-up**

**Table 2:** Clinical characteristics at baseline and at 13 years of follow up of the female participants with and without increase in serum uric acid. The 4^th^ (1994/95) and the 6^th^ (2007/08) Tromsø study (*n* = 1367)

	Baseline 1994/95 SUA non- increasers (*n* = 836)	SUA Increasers (*n* = 531)	*p*-value*	Follow-up 2007/08 SUA non- Increasers (*n* = 836)	SUA-increasers (*n* = 531)	*p*-value**
Age	57 ± 10	58 ± 9	0.17	70 ± 10	70 ± 9	0.17
SUA (μmol/L)	280 ± 67	253 ± 57	<0.001	264 ± 56	332 ± 74	<0.001
Estimated GFR (ml/min/1.73 m^2^)	95 ± 13	94 ± 14	0.35	85 ± 13	79 ± 18	<0.001
Annual change in estimated GFR (ml/min/1.73 m^2^)	-	-		−0.76 ± 0.72	−1.2 ± 0.99	<0.001
ACR (mg/mmol)	0.54 (0.38, 0.82)	0.56 (0.39,0.86)	0.42	0.48 (0.27, 0.87)	0.56 (0.29, 1.16)	0.01
Mean systolic blood pressure (mmHg)	138 ± 21	142 ± 23	0.002	148 ± 27	148 ± 25	1.0
Hypertension, n (%)	378 (45)	270 (51)	0.04	596 (71)	414 (77)	0.06
Body mass index (kg/m^2^)	25.5 ± 3.8	25.8 ± 4.1	0.09	26.3 ± 4.26	27.8 ± 4.8	<0.001
Cholesterol (mmol/L)	6.73 ± 1.34	6.68 ± 1.27	0.6	5.88 ± 1.07	5.95 ± 1.17	0.20
Current smokers, n (%)	216 (26)	147 (28)	0.45	142 (18)	65 (13)	0.02
Physical activity,n (%)	179 (21)	99 (17)	0.04	192 (23)	107 (20)	0.5
Antihypertensives incl. diuretics, n (%)	70 (8)	57 (11)	0.14	70 (8)	57 (10)	0.14
Diuretics, n (%)	6 (0.7)	7 (1.3)	0.3	44 (6)	96 (19)	<0.001
Diabetes, n (%)	14 (1.5)	15 (2.6)	0.15	75 (9)	60 (11)	0.2
Previous myocardial infarction and/or stroke, n (%)	25 (3)	6 (1)	0.002	-	-	-
Started antihypertensive treatment during the observation time, n (%)	-	-	-	230 (28)	212 (46)	<0.001
Stopped smoking during the observation time, n (%)	-	-	-	85 (10)	86 (16)	0.001
Became physically active during the observation time, n (%)	-	-	-	126 (15)	71 (13)	0.4

Values presented as in Table 1. ACR: urinary albumin/creatinine ratio. *P*-values for differences between the SUA increasers and non-increasers at baseline* and follow-up**

### Multivariable analyses

**Fig. 1:**
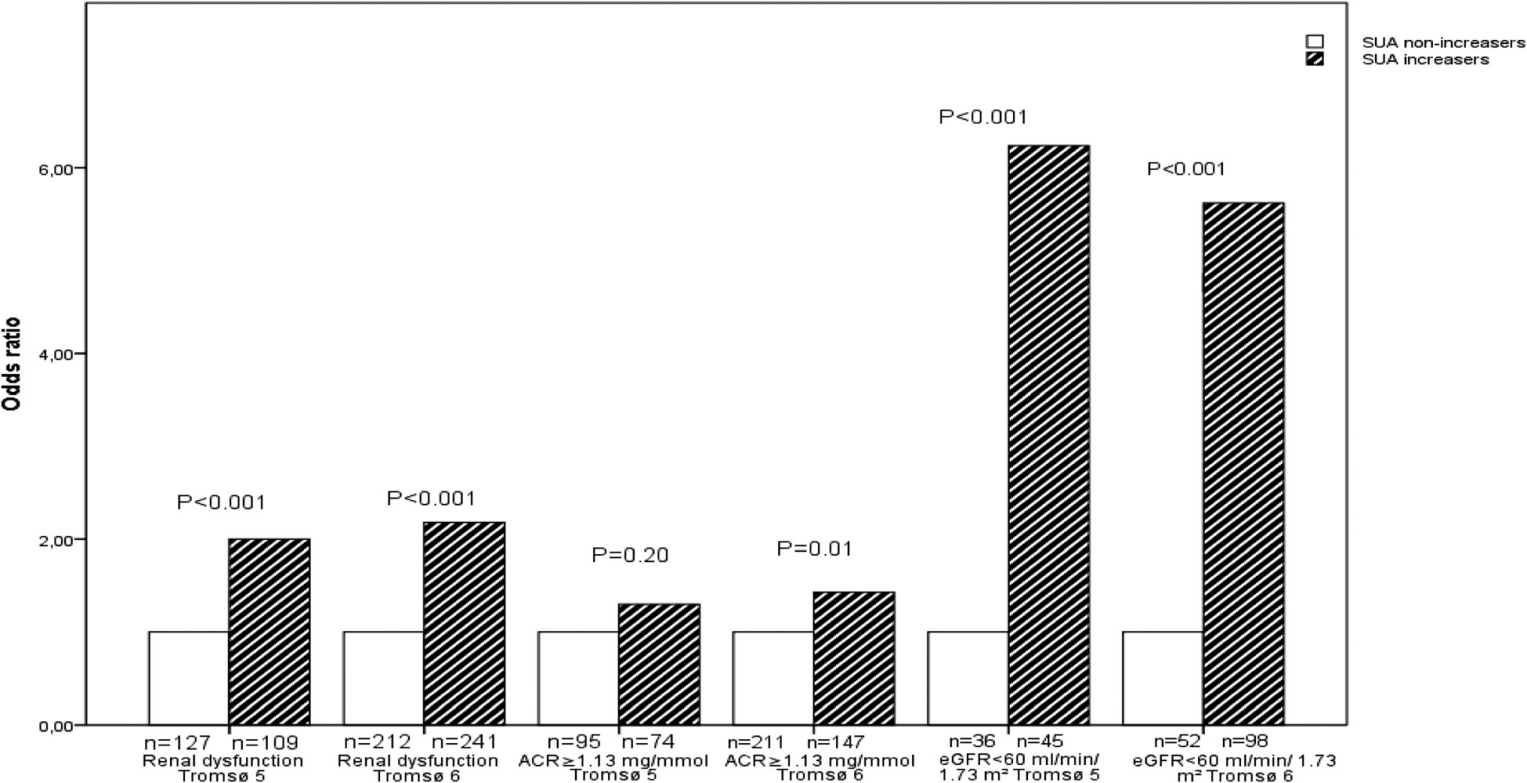
Odds ratios for renal dysfunction and its components after 7 and 13 years of follow-up in serum uric acid (SUA) increasers compared to SUA non-increasers. Only participants without renal dysfunction at baseline (*n* = 2215) are included

The results from the multiple logistic regression analyses with the dependent variables measured at 7 and 13 years of follow-up are presented in Fig. 1 and in Table 3. In the analyses presented in Fig. 1, only participants without RD at baseline were included, whereas all participants were included in Table 3. When the 422 participants with RD at baseline were excluded, an increase in baseline SUA of 59 μmol/L (corresponds to 1 mg/dL) gave an odds ratio (OR) of RD in Tromsø 6 of 1.16 (95 % confidence interval (CI) 1.04, 1.29). Being a SUA-increaser gave an OR for RD after 7 years of 2.00 (95 % CI 1.45, 2.75). The corresponding OR for RD after 13 years was 2.18 (95 % CI 1.71, 2.79). Among these participants, being a SUA increaser was not significantly associated with development of albuminuria after 7 years, but gave an OR for ACR ≥1.13 mg/mmol after 13 years of 1.43 (95 % CI 1.09, 1.86).

**Table 3:** Odds ratios for having renal dysfunction after 7 and 13 years of observation when participants with baseline RD were not excluded (*n* = 2637)

Risk factors	Renal dysfunction° Tromsø 5 2001/02 (*n* = 474)	*p*-value	ACR ≥ 1.13 mg/mmol Tromsø 5 2001/02 (*n* = 394)	*p*- value	Estimated GFR <60 ml/min/1.73 m^2^ Tromsø 5, 2001/02 (*n* = 117)	*p*- value
Age, per 5 years increase	1.13 (1.02, 1.25)	0.02	1.19 (1.06, 1.33)	0.002	1.44 (1.15, 1.80)	<0.001
SUA increasers^a^	1.98 (1.52, 2.59)	<0.001	1.35 (1.018, 1.82)	0.044	4.85 (2.87, 8.19)	<0.001
Baseline SUA, per 59 μmol/L (1 mg/dl) increase	1.10 (0.99, 1.24)	0.09	1.05 (0.92, 1.18)	0.5	1.42 (1.15, 1.75)	0.001
Estimated GFR per 5 ml/1.73 m2 decrease	1.22 (1.17, 1.27)	<0.001	1.05 (0.99, 1.12)	0.16	1.44 (1.38, 1.49)	<0.001
Log ACR per unit	26.9 (18.3, 39.7)	<0.001	44.87 (29.57, 68.08)	<0.001	1.47 (0.86, 2.49)	0.16
	Renal dysfunction Tromsø 6 2007/08 (*n* = 697)		ACR ≥ 1.13 mg/mmol Tromsø 6 2007/08 (*n* = 589)		Estimated GFR < 60 ml/min/1.73 m^2^ Tromsø 6, 2007/08 (*n* = 220)	
Age, per 5 years increase	1.27 (1.17, 1.38)	<0.001	1.31 (1.19, 1.43)	<0.001	1.65 (1.39, 1.94)	<0.001
SUA increasers^a^	2.12 (1.71, 2.65)	<0.001	1.46 (1.16, 1.85)	<0.001	5.11 (3.45, 7,56)	<0.001
Baseline SUA, per 59 μmol/L (1 mg/dl) increase	1.15 (1.05, 1.26)	0.003	1.09 (0.99, 1.21)	0.09	1.35 (1.16, 1.58)	<0.001
Estimated GFR per 5 ml/1.73 m^2^ decrease	1.11 (1.06, 1.16)	<0.001	0.98 (0.92, 1.03)	0.5	1.38 (1.33, 1.43)	<0.001
Log ACR per unit	6.99 (5.16, 9.48)	<0.001	11.11(8.05, 15.32)	<0.001	1.60 (1.06, 2.41)	0.03

*ACR* urinary albumin/creatinine ratio, *SUA* serum uric acid. °Renal dysfunction was defined as ACR ≥1.13 mg/mmol and/or estimated GFR <60 ml/min/1.73 m^2^. ^a^SUA increasers: the upper tertile of change in SUA from baseline to follow-up, compared to the two lower tertiles. Multivariate adjustments were performed for systolic blood pressure, BMI, cholesterol, current smoking; physical activity, antihypertensive drugs included diuretics, diabetes, previous myocardial infarction and/or stroke and change in systolic blood pressure during follow-up. We also included start of antihypertensive treatment during observation, cessation of smoking during observation or becoming physically active during observation as independent variables

When the entire cohort was studied (i.e. with the subjects with baseline RD included), we found that SUA increasers had a doubled risk of RD after 7 years. Being a SUA-increaser also was significantly associated with both the single components of the RD, ACR ≥ 1.13 mg/mmol and decreased eGFR < 60 ml/min/1.73 m^2^. After 13 years, the SUA increasers had an OR for RD of 2.12 (95 % CI 1.71, 2.65), and the risk of each component of the RD was also increased (Table 3). Baseline SUA was not a risk factor for increased ACR after 7 and 13 years, nor for RD after 7 years, whether the participants with baseline RD were excluded or not. However, baseline SUA was a significant predictor for the combined endpoint RD and for reduced eGFR after 13 years in both cases. Other baseline risk factors for RD after 7 and 13 years of follow-up were age, BMI, change in blood pressure, and current smoking. The participants who started with antihypertensive treatment had an increased risk for RD after 13 years. When we investigated baseline SUA as a predictor without adjusting for longitudinal change in SUA, the associations with the renal endpoints were not significant.

## Discussion

In this prospective study from a large cohort from the general population we found that being in the highest tertile of SUA change, corresponding to an increase in SUA of more than 33 μmol/L over 13 years, was an independent risk factor for RD defined as slightly increased ACR and/or a moderately reduced eGFR. This result was consistent whether the population had a long time follow-up of 13 years or whether the follow-up time was shorter. Moreover, the results were similar when participants with baseline RD were excluded. Although the OR for moderately reduced eGFR was higher than the OR for ACR ≥1.13 mg/mmol, longitudinally increasing SUA was significantly associated also with the development of albuminuria after 13 years. The associations between baseline SUA and the renal endpoints were not significant when the longitudinal change in SUA was not adjusted for. The reason for this is unclear, but it is possible that the association between baseline SUA and the renal endpoints within the SUA increaser-and non-increaser groups, respectively, becomes obscured when the whole cohort is studied without this group division. In addition, change in SUA can partly be explained as a regression to the mean phenomenon, and by adjusting baseline SUA for longitudinal change in the same variable, the influence of random outliers may have been reduced. Thus, it may be important to evaluate change in SUA in addition to one single raised value when considering the impact of SUA on renal endpoints. In our study, SUA increase was associated with worsening eGFR and ACR over time, and these two markers independently predict advanced stage CKD, cardiovascular disease and mortality [15–24].

Numerous reports suggest SUA as a risk factor for CKD and cardiovascular disease [2, 4, 25–28]. The largest study evaluated 177,570 patients in the US Renal Data System (USRDS), where the subjects were followed over 24 years [10]. Patients in the highest quartile of SUA had a hazard ratio of 2.14 for CKD. Obermayr et al. followed 21,475 healthy volunteers for 7 years [2]. After adjustment for baseline eGFR, a slightly elevated SUA level (7–8.9 mg/dl) was associated with a nearly doubled risk of incident CKD (eGFR <60 ml/min per 1.73 m^2^) and an elevated SUA of more than 9 mg/dl was associated with a tripled risk. This increased risk remained significant even after adjustment for baseline eGFR, gender, age, antihypertensive drugs, and components of the metabolic syndrome. In the Atherosclerosis Risks in Communities (ARIC) Study and the Cardiovascular Health Study [11], Weiner et al. pooled two community-based cohorts from the US. Each increase of 1 mg/dL in SUA was associated with a 7–11 % increase in incident of CKD. Weiner et al. point out two main limitations in their study: lack of information on baseline proteinuria and allopurinol use. In the study by Obermayr et al. [22], proteinuria was included. However, neither of them included ACR in their analyses. In the Tromsø Study, three urine specimens were collected and the mean ACR value was used, reducing the impact of the day-to-day variation. Also, the use of allopurinol was registered.

However, there were few allopurinol-users included in the study. Moreover; the use of allopurinol was not a significant predictor in the univariable analyses and therefore not included in the multivariable models.

In a recently published meta-analysis that included fifteen unique cohorts, the investigators demonstrated a positive association between SUA levels and the risk of CKD, defined as eGFR <60 mL/min/1.73 m^2^ at the follow-up examination, in middle-aged patients, independent of established metabolic risk factors. They conclude that future randomized, high-quality clinical trials are warranted to determine whether lowering SUA levels is beneficial in CKD [29]. Our study is in concordance with the results from this meta-analysis. However, the positive association in the meta-analysis was more pronounced among groups with a mean age < 60 years, and no association was observed in cohorts with a mean age ≥60 years. The present study included participants in a wide range of age, from 25 to 74 years, but the mean age at baseline was 56 years. Thus, we found an association among participants who were older than the cohorts included in the meta-analysis, and it is possible that our results would have been stronger if mainly younger persons were investigated.

Most studies use eGFR <60 mL/min/1.73 m^2^ as the end-point when considering CKD. We also included ACR ≥ 1.13 mg/mmol as an outcome, and showed that increasing SUA also predicted lowgrade albuminuria, although this association was slightly weakened when participants with baseline RD were excluded. According to the 2012 Kidney Disease Improving Global Outcome (KDIGO) CKD classification, we classified persons with “high normal” ACR (ACR ≥ 1.13 mg/mmol), as having RD. We chose this cut-off value since ACR of this magnitude has been shown to predict cardiovascular disease and mortality in the general population [18, 19].

To explain our findings on a basic biological level, several studies have investigated the effect of SUA on animals. Johnson et al. developed a model of hyperuricemia in rats by providing an uricase inhibitor [30]. The hyperuricemic rats developed subtle renal injury associated with activation of the reninangiotensin system and development of hypertension. Considering human biology, an interesting biopsy study was recently performed [31]. The association between SUA level and renal arteriolar hyalinosis and wall thickening in renal biopsies was assessed in a cross-sectional study of 167 CKD patients. As the SUA level increased, the degree of hyalinosis and wall thickening worsened.

The strengths of the present study were the prospective design, a large cohort from the general population with a high attendance rate, and a long observation time (13 years). We were able to adjust for the use of diuretics and other medications such as antihypertensive drugs and allopurinol. We also had ACR measurements from three unfrozen urine specimens available. In addition, our study was able to adjust for baseline eGFR, increasing the likelihood that the RD was a result of increasing SUA and not the other way around. However, because SUA change was calculated as the difference between the SUA values at baseline and the last follow-up, when also the outcome (RD) was assessed simultaneously, inverse causality between SUA change and decreasing eGFR is still a possibility.

Other limitations of the present study include the observational design, which precludes firm conclusions about causal inferences. As described earlier, only persons with three SUA measurements were included in the cohort. Analyses showed that the excluded persons were less healthy at baseline, and this may have influenced the results of the study. However, it is reasonable to believe that the inclusion of these less healthy individuals would have strengthened rather than weakened the reported associations. The population included in the Tromsø Study was mainly Caucasian. The results may therefore not be generalizable to other populations. However, increasing number of reports points to SUA as a risk factor for CKD in various populations including Asians [7, 8, 26, 27, 32, 33].

## Conclusion

In conclusion, the present prospective study shows for the first time that longitudinally increasing SUA is associated with RD, and for each individual components of RD, i.e. moderately decreased eGFR and slightly increased ACR. Our study also supports the growing evidence that SUA is a significant risk factor for CKD. Randomized controlled trials studying SUA decreasing agents are desired to finally decide whether asymptomatic hyperuricemia should be treated to reduce the incidence of RD also in low-risk populations.

## Abbreviations

GFR: Glomerular filtration rate
eGFR: estimated glomerular filtration rate
ACR: urinary albumin creatinine ratio
RD: renal dysfunction
SUA: serum uric acid
KDIGO: kidney disease improving global outcome
CKD: chronic kidney disease
BMI: body mass index

## References

[1] Levey AS, Atkins R, Coresh J, Cohen EP, Collins AJ, Eckardt KU (2007). Chronic kidney disease as a global public health problem: approaches and initiatives - a position statement from Kidney Disease Improving Global Outcomes. Kidney Int.

[2] Obermayr RP, Temml C, Gutjahr G, Knechtelsdorfer M, Oberbauer R, Klauser-Braun R (2008). Elevated uric acid increases the risk for kidney disease. J Am Soc Nephrol.

[3] Matsushita K, van der Velde M, Astor BC, Woodward M, Levey AS, de Jong PE (2010). Association of estimated glomerular filtration rate and albuminuria with all-cause and cardiovascular mortality in general population cohorts: a collaborative metaanalysis. Lancet.

[4] Filiopoulos V, Hadjiyannakos D, Vlassopoulos D (2012). New insights into uric acid effects on the progression and prognosis of chronic kidney disease. Ren Fail.

[5] Akasaka H, Yoshida H, Takizawa H, Hanawa N, Tobisawa T, Tanaka M (2014). The impact of elevation of serum uric acid level on the natural history of glomerular filtration rate (GFR) and its sex difference. Nephrol Dial Transplant.

[6] Jalal DI, Chonchol M, Chen W, Targher G (2013). Uric acid as a target of therapy in CKD. Am J Kidney Dis.

[7] Iseki K, Oshiro S, Tozawa M, Iseki C, Ikemiya Y, Takishita S (2001). Significance of hyperuricemia on the early detection of renal failure in a cohort of screened subjects. Hypertens Res.

[8] Domrongkitchaiporn S, Sritara P, Kitiyakara C, Stitchantrakul W, Krittaphol V, Lolekha P (2005). Risk factors for development of decreased kidney function in a southeast Asian population: a 12-year cohort study. J Am Soc Nephrol.

[9] Iseki K, Ikemiya Y, Inoue T, Iseki C, Kinjo K, Takishita S (2004). Significance of hyperuricemia as a risk factor for developing ESRD in a screened cohort. Am J Kidney Dis.

[10] Hsu CY, Iribarren C, McCulloch CE, Darbinian J, Go AS (2009). Risk factors for end-stage renal disease: 25-year follow-up. Arc Intern Med.

[11] Weiner DE, Tighiouart H, Elsayed EF, Griffith JL, Salem DN, Levey AS (2008). Uric acid and incident kidney disease in the community. J Am Soc Nephrol.

[12] Jacobsen BK, Eggen AE, Mathiesen EB, Wilsgaard T, Njolstad I (2012). Cohort profile: the Tromso Study. Int J Epidemiol.

[13] Levey AS, Stevens LA, Schmid CH, Zhang YL, Castro AF, Feldman HI (2009). A new equation to estimate glomerular filtration rate. Ann Intern Med.

[14] Levey AS, Coresh J (2012). Chronic kidney disease. Lancet.

[15] Chen YY, Li YY, Lu YH, Dou JT, Wang SY, Lu JM (2012). Albuminuria independently predicts cardiovascular and all-cause mortality in a middle-aged and elderly Chinese population. Scand J Clin Lab Invest.

[16] Bello A, Thompson S, Lloyd A, Hemmelgarn B, Klarenbach S, Manns B (2012). Multiple versus single and other estimates of baseline proteinuria status as predictors of adverse outcomes in the general population. Am J Kidney Dis.

[17] Arnlov J, Evans JC, Meigs JB, Wang TJ, Fox CS, Levy D (2005). Low-grade albuminuria and incidence of cardiovascular disease events in nonhypertensive and nondiabetic individuals: the Framingham Heart Study. Circulation.

[18] Romundstad S, Holmen J, Kvenild K, Hallan H, Ellekjaer H (2003). Microalbuminuria and all-cause mortality in 2,089 apparently healthy individuals: a 4.4-year follow-up study. The NordTrondelag Health Study (HUNT), Norway. Am J Kidney Dis.

[19] Stephen R, Jolly SE, Nally JV, Navaneethan SD (2014). Albuminuria: when urine predicts kidney and cardiovascular disease. Cleve Clin J Med.

[20] Holzmann MJ, Aastveit A, Hammar N, Jungner I, Walldius G, Holme I (2012). Renal dysfunction increases the risk of ischemic and hemorrhagic stroke in the general population. Ann Med.

[21] Gansevoort RT, Matsushita K, van der Velde M, Astor BC, Woodward M, Levey AS (2011). Lower estimated GFR and higher albuminuria are associated with adverse kidney outcomes. A collaborative meta-analysis of general and high-risk population cohorts. Kidney Int.

[22] Cheng TY, Wen SF, Astor BC, Tao XG, Samet JM, Wen CP (2008). Mortality risks for all causes and cardiovascular diseases and reduced GFR in a middle-aged working population in Taiwan. Am J Kidney Dis.

[23] Astor BC, Hallan SI, Miller ER, Yeung E, Coresh J (2008). Glomerular filtration rate, albuminuria, and risk of cardiovascular and all-cause mortality in the US population. Am J Epidemiol.

[24] Verhave JC, Gansevoort RT, Hillege HL, Bakker SJ, De Zeeuw D, de Jong PE (2004). An elevated urinary albumin excretion predicts de novo development of renal function impairment in the general population. Kidney Int Suppl.

[25] Liu WC, Hung CC, Chen SC, Yeh SM, Lin MY, Chiu YW (2012). Association of hyperuricemia with renal outcomes, cardiovascular disease, and mortality. Clin J Am Soc Nephrol.

[26] Yamada T, Fukatsu M, Suzuki S, Wada T, Joh T (2011). Elevated serum uric acid predicts chronic kidney disease. Am J Med Sci.

[27] Sonoda H, Takase H, Dohi Y, Kimura G (2011). Uric acid levels predict future development of chronic kidney disease. Am J Nephrol.

[28] Katsiki N, Karagiannis A, Athyros VG, Mikhailidis DP (2013). Hyperuricaemia: more than just a cause of gout?. J Cardiovasc Med.

[29] Zhu P, Liu Y, Han L, Xu G, Ran JM (2014). Serum uric acid is associated with incident chronic kidney disease in middle-aged populations: a meta-analysis of 15 cohort studies. PLoS One.

[30] Johnson RJ, Nakagawa T, Jalal D, Sanchez-Lozada LG, Kang DH, Ritz E (2013). Uric acid and chronic kidney disease: which is chasing which?. Nephro Dial Transplant.

[31] Kohagura K, Kochi M, Miyagi T, Kinjyo T, Maehara Y, Nagahama K (2013). An association between uric acid levels and renal arteriolopathy in chronic kidney disease: a biopsy-based study. Hypertens Res.

[32] Wang S, Shu Z, Tao Q, Yu C, Zhan S, Li L (2011). Uric acid and incident chronic kidney disease in a large health check-up population in Taiwan. Nephrology (Carlton).

[33] Okada Y, Sim X, Go MJ, Wu JY, Gu D, Takeuchi F (2012). Meta-analysis identifies multiple loci associated with kidney function-related traits in east Asian populations. Nat Genet.

